# Preliminary Schools for Probationers

**Published:** 1909-03-27

**Authors:** 


### Nursing Administration.

### PRELIMINARY SCHOOLS FOR PROBATIONERS.

### III.—THE COST.

The ordinary rate of charge for instruction in nursing in institutions is one guinea a week. Hospitals giving a shortened form of nursing instruction to pupil nurses, lasting three, six, or twelve months, commonly make this charge, designed to cover the expense of board, lodging and tuition. It offers, of course, bare remuneration to the hospital for the expenses of paying probationers, although these individuals, or more commonly their relatives, appear sometimes to labour under the impression that the hospital makes a large profit out of their fees and services combined. In the case of the preliminary school, it will require very careful management to make both ends meet by charging a guinea a week, but experience both in London and the provinces shows that it can be done. It is necessary however to consider the fee in the first instance from the point of view of the pupil. Is it prohibitive in its effects, and can a sufficient choice of probationers be secured when what amounts to a premium of six or eight guineas is obligatory on the candidate? It would seem at first sight as though the hospital would run some risk in introducing this paying course as a passport to service in the institution. Premiums for probationers have now almost completely fallen into disuse, and for this reason, that the candidates willing to pay are found by matrons to fall below the standard of those who can be obtained when no premium is required. Too often it happens that those prepared to pay for their first year of probationership are those who have tried in vain to secure for themselves free training in other institutions. They represent the "leavings." But this only happens because of late years there are so many excellent training schools where no premium is asked, and where payment is given from the beginning. Few sensible people are so constituted as to desire to pay in one place for what can be had free of charge in another. And the hospitals requiring premiums altogether failed to show that they could offer additional advantages for the fee received. In the case of the preliminary school, however, very conspicuous advantages are offered to the pupils. The thought of plunging headlong and unprepared into the stress of hospital work deters many possible probationers altogether from offering themselves as candidates. They know perhaps some friend who has broken down in the process, and they are keenly conscious of their own deficiencies. The prospect of learning their duties systematically before they begin to enter the wards attracts exactly the class of zealous and earnest-minded women who are wanted, and such women, however poor, will not shrink from testing their vocation by saving the amount of the necessary fees from a scanty salary. For a large proportion of the best nurses are not idle women looking for something to do, but are hard workers in some other calling before they begin their training at the age of 23. They will make a sacrifice of a few pounds, or their relatives will help them, if it is made clear to them that thereby they may advance their future career.

Will a guinea a week suffice to board and lodge, teach, and examine the pupils until they enter the institution as probationers? It depends entirely on the extent to which economy is practised in the rest of the institution. If the nurse's food is allowed to cost nine or ten shillings a week, and if other household expenses are allowed to run up in a corresponding manner, the thing cannot be done. But when inspection is thorough and the housekeeping is well in hand, the actual expense of boarding and lodging the pupil need not exceed ten shillings and sixpence a week. This is the amount spent on each pupil by Guy's Hospital; the amount not being "estimated," but demonstrated by figures. Six shillings a head weekly, represents the cost of excellent and varied diet, and this leaves four shillings a head for a due proportion of establishment charges. Probably if a separate house were provided for the pupils the cost would be at least double.

Deducting then the sum of ten shillings and sixpence for board, lodging, and other establishment expenses, there remains ten shillings a week each for the expenses of tuition, printing, postage, and examinations. It will be more convenient to treat this as a yearly sum. Supposing that the school contains eight pupils, and is filled and emptied seven times in the year, the fees from the pupils will amount to £6 6s. × 8 × 7 = £352 16s., each pupil paying £6 6s. for her course. Deduct half of this amount for board and lodging, and the remainder is £176 8s., to defray the following expenses:—

| | |
|---|---|
| Salary of sister in charge ... ... ... ... | £45 |
| Board and lodging of sister in charge ... | 26 |
| Half-salary of staff nurse ... ... ... | 17 |
| Servant's wages ... ... ... ... ... | 14 |
| Servant's board and lodging ... ... ... | 18 |
| Cookery teacher's fee, examiner's fee, postage and printing at an average of £8 per course ... ... ... ... ... ... ... | 56 |
| | £176 |

The estimate of expenses for tuition, etc., in each course is a good deal more in proportion than the cost of the school at Guy's, where the cost is £11 per course for the 15 or 16 pupils received. But in such items as teaching and examining, the cost does not diminish proportionally when numbers are reduced. Any saving which might be effected in this direction would be absorbed in additional establishment charges spread over the smaller number. The figures clearly demonstrate the fact that no deficit need be feared when the preliminary school is organised on practical lines. The pupils pay their own laundry expenses, and provide uniform at their own cost, the average expense of the uniform being £3 3s. The outside cost to the pupil is therefore £10.

